# Perspectives on a ‘Sit Less, Move More’ Intervention in Australian Emergency Call Centres

**DOI:** 10.3934/publichealth.2016.2.288

**Published:** 2016-05-19

**Authors:** Josephine Y Chau, Lina Engelen, Sarah Burks-Young, Michelle Daley, Jen-Kui Maxwell, Karen Milton, Adrian Bauman

**Affiliations:** 1 Prevention Research Collaboration, School of Public Health, The Charles Perkins Centre, University of Sydney; 2 National Heart Foundation of Australia, New South Wales Division; 3 British Heart Foundation Centre on Population Approaches for Non Communicable Disease Prevention, Nuffield Department of Population Health, University of Oxford

**Keywords:** Sedentary behaviour, workplace, shift-workers, qualitative, health promotion

## Abstract

**Background:**

Prolonged sitting is associated with increased risk of chronic diseases. Workplace programs that aim to reduce sitting time (sit less) and increase physical activity (move more) have targeted desk-based workers in corporate and university settings with promising results. However, little is known about ‘move more, sit less’ programs for workers in other types of jobs and industries, such as shift workers. This formative research examines the perceptions of a ‘sit less, move more’ program in an Australian Emergency Call Centre that operates 24 hours per day, 7 days per week.

**Methods:**

Participants were employees (N = 39, 72% female, 50% aged 36–55 years) recruited from Emergency Services control centres located in New South Wales, Australia. The ‘sit less, move more’ intervention, consisting of emails, posters and timer lights, was co-designed with the management team and tailored to the control centre environment and work practices, which already included electronic height-adjustable sit-stand workstations for all call centre staff. Participants reported their perceptions and experiences of the intervention in a self-report online questionnaire, and directly to the research team during regular site visits. Questionnaire topics included barriers and facilitators to standing while working, mental wellbeing, effects on work performance, and workplace satisfaction. Field notes and open-ended response data were analysed in an iterative process during and after data collection to identify the main themes.

**Results:**

Whilst participants already had sit-stand workstations, use of the desks in the standing position varied and sometimes were contrary to expectations (e.g, less tired standing than sitting; standing when experiencing high call stress). Participants emphasised the “challenging” and “unrelenting” nature of their work. They reported sleep issues (“always tired”), work stress (“non-stop demands”), and feeling mentally and physically drained due to shift work and length of shifts. Overall, participants liked the initiative but acknowledged that their predominantly sitting habits were entrenched and work demands took precedence.

**Conclusions:**

This study demonstrates the low acceptability of a ‘sit less, move more’ program in shift workers in high stress environments like emergency call centres. Work demands take priority and other health concerns, like poor sleep and high stress, may be more salient than the need to sit less and move more during work shifts.

## 1. Introduction

Sedentary behaviour is associated with an increased risk of chronic disease [1]–[4], with physical activity providing some protective effects to offset sitting risk [5]. Sedentary behaviour refers to activities that are done in a sitting or reclining posture and cost ≤ 1.5 times the basal metabolic rate, or the energy expended by the body at rest [6]. Sedentary behaviour is distinct from a lack of physical activity. It is possible for people to meet recommended physical activity levels (150 minutes of moderate to vigorous physical activity per week), yet spend large amounts of time sitting, particularly at work.

The World Health Organisation and World Economic Forum have highlighted the workplace as an important setting for health promotion [7]. Workers in desk-based occupations are considered a key target group for sitting reduction programs in the workplace [8], [9].

Workplace programs that aim to reduce sitting time (sit less) and increase physical activity (move more) have targeted desk-based workers in corporate and university settings with some showing promising results [10], [11]. Nevertheless, many earlier intervention studies have involved selected samples from health-related organisations or universities with tertiary level educations [12]–[17] and there is a knowledge gap about programs targeting occupational sitting and physical activity in workers in other types of jobs industries and work arrangements, such as shift workers.

Emergency call centre workers are a particular at-risk group for chronic ill health due to their prolonged sitting at work on account of the desk-based and computer-reliant nature of their work and low opportunity for movement away from their desks. Studies report that call centre operators spend 81–95% of their work shift in a seated posture [18], [19]. Additionally, emergency call centre work, characterized by shift work as well as the repetitive and stressful nature of work tasks, places extra burden on employee's physical and mental wellbeing [20]. Previous qualitative research among medical call operators in South Africa found employees reported feelings of stress due to the nature of the work itself, perceptions of being undervalued at work, organizational factors, and lack of support outside of work [21].

This formative research examines the perceptions of a ‘sit less, move more’ pilot program in an emergency call centre that operates 24 hours per day, 7 days per week. The purpose of this study is to gain insight into the experiences, feasibility and acceptability of a tailored sitting reduction intervention in a relatively understudied desk-based shift worker group. The aims of this study were to assess participants' perception of the ‘sit less, more more’ program, and to examine contextual factors affecting program acceptability such as other health issues and job demands.

## 2. Methods

### 2.1. Participants and design

Participants were employees working in two call centres for one emergency services organisation in New South Wales, Australia. The program was advertised to staff as a workplace wellness initiative and interested employees contacted the research team, who provided additional study information. Participants joined the study by signing and returning a consent form. Employees aged at least 18 years old and working at least three 12-hour shifts per 9-day rotation were eligible to join this research. This study took place from July to October in 2015 and was approved by the University of Sydney Human Research Ethics Committee (No. 2015/224). This qualitative report is one component of the program evaluation and details about the quantitative findings are reported elsewhere.

This study had a quasi-experimental design. The two call centres were assigned to an intervention and a control condition a priori by the partner organisation. The intervention was designed in collaboration with the organisation's health and wellness program manager and deputy director. All call centre staff already worked at electronically operated height-adjustable workstations. Although the workstations had been provided to staff for approximately 17 years, managers and senior officers noted that they were seldom used at standing height.

The ‘sit less, move more’ intervention consisted of three components: emails, timer lights, and posters. Weekly informative emails were sent to participants with material about prolonged sitting and health, and reminders to stand up more, as well as tips, infographics and a brief video (“Sit Less Move More Whiteboard Animation”, www.youtube.com/watch?v=juV1vnGoOQ4) about how to increase standing and sit less at work. Timer lights with the message “Try standing up” were mounted at two ends of the open plan call centre and set to an electronic timer that turned the lights on, illuminating the message, for 30 minutes and off for 60 minutes continuously. The lights turned on and off a total of 32 times per 24-hour period and were illuminated for eight 30-minute periods during a 12-hour shift. Participants were encouraged to use the timer light as a visual reminder to stand up during shift time. As participants were often taking phone calls for extended periods of time and expressed that they did not want to change their posture during a call, the light was used as a prompt to think about changing their posture in between calls. It was clearly explained to participants that the duration of the light being illuminated was not indicative of the amount of time that they should have spent sitting or standing but rather a visual reminder for them to stand more and break up their sitting. Heart Foundation of Australia ‘Sit less, move more’ posters also were displayed on notice boards in the call centre, kitchen and hallways. Control participants received the intervention resources after the evaluation was completed.

### 2.2. Procedures

The program was implemented over an 11-week period with baseline measures taken prior to program implementation, and follow-up measures at 5 weeks and 10 weeks after program implementation. The total duration of the intervention was 75 days (10.7 weeks). At baseline and at each follow-up, participants completed open-ended self-report online survey questions about their perceptions and experiences of the program. Open-ended questions asked about barriers and facilitators to standing while working, mental wellbeing, effects on work performance, and workplace satisfaction. Additionally, the research team had regular contact with participants over the course of the study via email and during face-to-face site visits and kept a log of field notes about conversations with participants and observations while on site.

### 2.3. Data analysis

We used a general qualitative method of analysis of all comments from participants and field notes gathered by online survey, email, and face-to-face during site visits in an iterative process during and after data collection. We used a predetermined coding framework based on study aims and survey topics to identify emerging ideas. Initial coding was conducted and summarised by one investigator (SB) and then reviewed by two other investigators independently (JYC, LE). The final results were determined through discussion and agreement by all three investigators who reviewed the data.

**Table 1.** Coding framework topics.

Main topic	Sub-topics
Sitting and standing at work	Facilitators, barriers, likes, dislikes, habits
Job satisfaction and productivity	Positives, negatives
Physical health	Musculoskeletal complaints, chronic conditions
Mental wellbeing	Positive and negative mood, stress
Other comments or observations	

## 3. Results

Thirty-nine employees provided qualitative data via the online survey: 72% were female, 50% were aged 36–55 years old, and mean years of service was 10.4 ± 9.5 years. One third (33.3%) of participants had been employed for 14 years or more at the organization. Of the 39 participants, 22 were from the intervention group and 17 were from the control group.

Here, we present the qualitative survey results from the 39 participants supplemented with data collected from email feedback (n = 2) and researchers' field notes. All quotations are taken from online responses with participants' study group identified. As control participants did not receive the intervention, they are not included in results about perceptions of the program and sitting and standing at work.

### 3.1. Perceptions about the program and sitting and standing at work

Overall, participants liked the program and appreciated the initiative from their managers to help them reduce and break up prolonged periods of sitting during shifts. They thought the program was “*fun*” (Participant 17, Intervention), “*very useful*” (Participant 36, Intervention), and “*a* *good distraction*” (Participant 14, Intervention). Nonetheless, participants acknowledged that their sit-stand habits were entrenched and work demands took precedence.

*“Great idea, but [it's] hard to break habits”* (Participant 34, Intervention).

The benefits of being able to alternate between sitting and standing on the job was acknowledged but perceptions with regard to implementing standing breaks into work routines varied. Some participants said it was difficult to work standing up, particularly on night shifts, while others found standing up to be less tiring and helpful for dealing with stressful calls. These positive comments attributed to standing were supported by observations from the floor manager who reported that call-takers tended to stand during periods of high call volume and stress and sit during quieter periods.

*“I don't feel I can concentrate as well when I am standing”* (Participant 34, Intervention).

*“Less tired standing rather than sitting too long”* (Participant 17, Intervention).

Social motivation was also cited by participants as helping them incorporate more standing into their work shift.

*“Other people standing made me stand more”* (Participant 3, Intervention).

Specific sit-stand strategies cited included having a buddy at work to remind each other to sit or stand, and having a champion on shift who took the initiative to call out to everyone on the floor that the reminder light for standing up had turned on. Several participants developed their own individual standing routines based on their break schedules (e.g., standing between breaks 1 and 2, then sitting between breaks 2 and 3).

During site visits, workers with longer tenure at the organisation revealed that the height-adjustable desks were originally commissioned in the late 1990s to accommodate call-takers and dispatchers of different heights so that they would be able to work in an ergonomically safe posture whilst sitting down. In other words, the height-adjustable desks were not originally installed to facilitate a sit-stand work style. Some of these longer-tenured workers expressed surprise and delight that the height-adjustable capacity of the desks could be reframed as a means for them to physically sit or stand up whilst on shift, while others saw this reframe as a diversion from call centre staff's other ergonomic needs and preferred wellness issues, such as the provision and maintenance of ergonomically safe chairs, and healthier food options in the kitchen.

### 3.2. Perceptions about other health issues

Participant's general health related comments underscore the impacts of shift work on their sleep and physical and mental health. Sleep was the predominant concern raised in regards to health. Participants said it was difficult to keep regular sleep patterns due to the nature of shift work and the scheduling of shifts. Irregular sleep patterns in turn led to mental and physical fatigue, such that participants reported feeling tired frequently. Insufficient staffing was also mentioned as a contributing factor to poorer mental wellbeing as this resulted in higher call handling volume and fewer breaks during shift. Few participants mentioned physical complaints, and those who did mentioned pain in the neck, back, knees, and joints, and attributed these complaints to activities both at work and outside of work.

*“I am constantly on call so the amount of sleep I get varies”* (Participant 54, Control).

*“Hard to gauge sleeping patterns due to shift work and not a long time between shifts”* (Participant 69, Intervention).

*“It is a mentally draining job that affects MH [mental health]. [It is] physically draining due to length of shift and shift work in general”* (Participant 17, Intervention).

### 3.3. Perceptions about job satisfaction and productivity

The majority of participant comments emphasized the busy and high pressure nature of their jobs, characterizing their work as “unrelenting”, “challenging”, and “time critical”. In contrast, some participants perceived their work as “boring” and “monotonous”, while others highlighted the challenges of dealing with constant disruptions and the impact of this on their productivity. There was also a general perception of being undervalued among participants; they felt they were not recognized nor rewarded for their work, which is critical to the health and safety of the public, and also extremely stressful and demanding.

*“You answer every call you have no choice to not answer — you answer within 3 rings — you read the script verbatum [sic] — you listen and care about the client — you are assessed on your customer service and you have every call recorded for customer service, professional conduct and legal considerations — it is just a statement of fact kind of job”* (Participant 13, Intervention).

*“Due to the work we do we are constantly responding to Stimuli of a time critical nature. So it's stimulating in this way but also tiring because of the nonstop demands. Towards the end of the day (almost every day) the quality of my work and enthusiasm will wane”* (Participant 3, Intervention).

There was a sense that negative perceptions of work were the norm, such that those people with more positive views and goals were considered exceptions. Positive perceptions of work and job satisfaction were characterized by a sense of rising to the challenge.

*“I'm an exception, but I love my job and the challenges it brings — I work to not get bogged down by the negativity of dealing long term with the public at their worst and strive to bring satisfaction and enjoyment to myself and those I work with”* (Participant 64, Control).

## 4. Discussion

This research examined the perceptions of emergency call centre workers about a tailored ‘sit less, move more’ pilot program in their workplace. These findings provide insights into a relatively understudied desk-based workforce that operates in shifts 24 hours per day, 7 days per week in a high stress environment. Overall, participants found this ‘sit less, move more’ program acceptable and enjoyable but highlighted the challenging and unrelenting nature of emergency call centre work as more salient demands. While they acknowledged the benefits of being able to sit and stand during work, sleep and work stress were also causes of concern and sources of mental and physical fatigue.

All call centre staff in this emergency services organization already had electronically operated height-adjustable workstations. Managers noted that the workstations were underutilized in the standing position and worked with the research team to design a program to promote less sitting and more standing tailored to the emergency call centre environment and work practices. The lack of workstation use at standing height was best explained by longer-tenured staff who had been with the organization since the late 1990s, when the height-adjustable workstations were installed. Originally, the height-adjustable capacity of the desks was for the purposes of accommodating call-takers and dispatchers of different heights so that they would be able to work in an ergonomically safe posture whilst seated and not to facilitate a sit-stand work style. The more recent experience of office workers in an Australian government organization shows similarities; management procured new sit-stand desks for staff but desk use in the standing position varied widely among staff because they had received no information about the new sit-stand desks, nor why and how they might wish to vary between sitting and standing during work time [22].

The co-designed nature of this program was implemented from the top down, rather than bottom up, and may have influenced staff perceptions of the program. This is demonstrated by the view from some participants that this program was a distraction from more pressing occupational health and safety needs, such as the procurement of new chairs and the maintenance of existing office furniture. Previous qualitative work in a health-related non-government organisation found more positive responses and greater sit-stand desk use uptake from staff when they had more engagement in organizational policy and practice [23]. It is the responsibility of both employees and managers to implement workplace health programs [24], and in sit-stand desk studies, greater uptake of standing to work can be achieved through investment in education and support for employees to change their sit-stand habits [25]. However, the managers who co-designed this program were familiar with employees' work demands and culture and as such were well placed to advise on program components for a ‘sit less, move more’ program. Participants' low engagement with the program is an indication of managers' misunderstanding of employees' health needs and priorities.

Participants reported sitting or standing to work in contrasting scenarios. Sitting and standing were useful for coping with the fatigue and stress of work, and they also acted as facilitators and barriers to completing work tasks in different workers, in line with previous findings [22]. Some participants indicated sitting to work during periods of high call volume and stress, while others reported standing up during these times; some participants said fatigue was a barrier to standing up during the shift, while others said standing made them feel less tired. Time-based strategies appeared to be less popular, with only a few participants reporting varying their sit-stand behaviour based on the timer lights or rest break schedule. Similar to previous findings [23], social flow-on effects were noted and an atmosphere of collegiality was observed during some shifts.

It is evident that participants felt they had autonomy over their postural allocation and could freely choose to sit or stand as they pleased during work shifts. The perceived choice whether to sit or stand aligns well with the views of Australian occupational health and safety practitioners, who have stressed that standing to work should be considered optional for employees rather than as compulsory, to remove any potential for perceived coercion [26]. Likewise, executives from Belgian companies have also stated a preference for employees to have choice regarding how and when they might sit less [27] While reducing and disrupting prolonged periods of sitting have potential health benefits [3], [28], there may also be harms associated with prolonged standing, such as musculoskeletal complaints and varicose veins [29], [30]. Therefore, any ‘sit less, move more’ workplace program should avoid encouraging employees to stand up all day at work and instead encourage staff to alter their posture and move around as much as possible.

These results highlight the importance of formative research when developing new workplace programs in real world settings. Whilst management regarded call centre employee's high levels of prolonged sitting as a health and wellbeing issue, participant feedback suggests that staff sleep patterns or stress management were higher priorities and in need of urgent attention. Unlike other sit-stand desk studies in office-workers, musculoskeletal complaints were seldom mentioned by emergency call centre workers in this study and suggest other health issues, like sleep and stress, were more salient. It was a limitation that this ‘sit less, move more’ program was designed and implemented from the top down with little consultation with the employees themselves. A more collaborative approach driven by managers and employees may have yielded greater participant engagement [22], [23].

A strength of this study is the involvement of emergency call centre workers, a group rarely studied in the sedentary behaviour workplace intervention field, which has largely focused on white collar office workers [12]–[17], [22]. Emergency call centre workers comprise a difficult to reach workforce, as shift workers in a high stress call centre environment with little flexibility for movement away from their workstations. These findings would be relevant to other emergency, shift-based, or high stress call centre workplaces, but less generalizable to office workers in corporate settings.

## 5. Conclusions

This study shows the low acceptability of a ‘sit less, move more’ program in shift workers in high stress environments like emergency call centres, as well as the importance of co-designing interventions with organisational partners. The 17-year provision of sit-stand workstations in this workplace demonstrates that providing sit-stand workstations alone is not sufficient for reducing sitting in emergency call centre workers and other ‘sit less, move more’ strategies should be considered. However, work demands take precedence and other health concerns, like poor sleep and high stress, may be more salient than the need to sit less and move more during work shifts.

## References

[1] Proper KI, Singh AS, van Mechelen W (2011). Sedentary behaviors and health outcomes among adults: a systematic review of prospective studies. Am J Prev Med.

[2] Wilmot EG, Edwardson CL, Achana FA (2012). Sedentary time in adults and the association with diabetes, cardiovascular disease and death: systematic review and meta-analysis. Diabetologia.

[3] Bauman AE, Chau JY, Ding D (2013). Too much sitting and cardio-metabolic risk: an update of epidemiological evidence. Curr Cardio Risk Rep.

[4] Van Uffelen JG, Wong J, Chau JY (2010). Occupational sitting and health risks: a systematic review. Am J Prev Med.

[5] Chau JY, Grunseit A, Chey T (2013). Daily sitting time and all-cause mortality: a meta-analysis. PLoS One.

[6] Sedentary Behavior Research Network (2012). Standardized use of the terms “sedentary” and “sedentary behaviours”. Appl Physiol Nutr Metab.

[7] WHO/WEF (2008). Preventing Noncommunicable Diseases in the Workplace Through Diet and Physical Activity: WHO/World Economic Forum Report of a Joint Event.

[8] Gilson N, Straker L, Parry S (2012). Occupational sitting: practitioner perceptions of health risks, intervention strategies and influences. Health Promotion J Australia.

[9] Thorp AA, Healy GN, Winkler E (2012). Prolonged sedentary time and physical activity in workplace and non-work contexts: a cross-sectional study of office, customer service and call centre employees. Int J Behav Nutr Phys Act.

[10] Neuhaus M, Eakin EG, Straker L (2014). Reducing occupational sedentary time: a systematic review and meta-analysis of evidence on activity-permissive workstations. Obes Rev.

[11] Shrestha N, Ijaz S, Kukkonen-Harjula KT (2015). Workplace interventions for reducing sitting at work. Cochrane Database of Systematic Reviews.

[12] Gilson ND, Suppini A, Ryde GC (2012). Does the use of standing ‘hot’ desks change sedentary work time in an open plan office?. Prev Med.

[13] Healy GN, Eakin EG, LaMontagne AD (2013). Reducing sitting time in office workers: Short-term efficacy of a multicomponent intervention. Prev Med.

[14] Pronk NP, Katz AS, Lowry M (2012). Reducing occupational sitting time and improving worker health: the take-a-stand project, 2011. Prev Chronic Dis.

[15] Neuhaus M, Healy GN, Dunstan DW (2014). Workplace sitting and height-adjustable workstations: a randomized controlled trial. Am J Prev Med.

[16] Cooley D, Pedersen S (2013). A pilot study of increasing nonpurposeful movement breaks at work as a means of reducing prolonged sitting. J Environ Public Health.

[17] Chau JY, Daley M, Dunn S (2014). The effectiveness of sit-stand workstations for changing office workers' sitting time: results from the Stand@ Work randomized controlled trial pilot. Int J Behav Nutr Phys Act.

[18] Straker L, Abbott RA, Heiden M (2013). Sit-stand desks in call centres: Associations of use and ergonomics awareness with sedentary behavior. Appl Ergon.

[19] Toomingas A, Forsman M, Mathiassen SE (2012). Variation between seated and standing/walking postures among male and female call centre operators. BMC Public Health.

[20] Brum MCB, Filho FFD, Schnorr CC (2015). Shift work and its association with metabolic disorders. Diabetology & Metabolic Syndrome.

[21] Lutrin J (2006). Identifying medical call centre stress: An evaluation of psychological and physical wellbeing. PhD Dissertation.

[22] Grunseit AC, Chau JY, van der Ploeg HP (2013). “Thinking on your feet”: A qualitative evaluation of sit-stand desks in an Australian workplace. BMC Public Health.

[23] Chau JY, Daley M, Srinivasan A (2014). Desk-based workers' perspectives on using sit-stand workstations: a qualitative analysis of the Stand@ Work study. BMC Public Health.

[24] Gilson ND, Burton NW, Van Uffelen JG (2011). Occupational sitting time: employees' perceptions of health risks and intervention strategies. Health Promotion J Australia.

[25] Wilks S, Mortimer M, Nylén P (2006). The introduction of sit-stand worktables: aspects of attitudes, compliance and satisfaction. Appl Ergon.

[26] Gilson N, Straker L, Parry S (2012). Occupational sitting: practitioner perceptions of health risks, intervention strategies and influences. Health Promotion J Australia.

[27] De Cocker K, Veldeman C, De Bacquer D (2015). Acceptability and feasibility of potential intervention strategies for influencing sedentary time at work: focus group interviews in executives and employees. Int J Behav Nutr Phys Act.

[28] Dunstan DW, Kingwell BA, Larsen R (2012). Breaking up prolonged sitting reduces postprandial glucose and insulin responses. Diabetes Care.

[29] Halim I, Rahman A, Saman AM (2012). Assessment of muscle fatigue associated with prolonged standing in the workplace. Saf Health Work.

[30] Tuchsen F, Hannerz H, Burr H (2005). Prolonged standing at work and hospitalisation due to varicose veins: a 12 year prospective study of the Danish population. Occup Environ Med.

